# Scandium Radioisotopes—Toward New Targets and Imaging Modalities

**DOI:** 10.3390/molecules28227668

**Published:** 2023-11-19

**Authors:** Krzysztof Kilian, Krystyna Pyrzyńska

**Affiliations:** 1 Heavy Ion Laboratory, University of Warsaw, Pasteura 5a, 02-093 Warsaw, Poland; 2 Faculty of Chemistry, University of Warsaw, Pasteura 1, 02-093 Warsaw, Poland; kryspyrz@chem.uw.edu.pl

**Keywords:** theranostics, scandium radioisotopes, radiolabeling, radiopharmaceuticals, radiochemical separation

## Abstract

The concept of theranostics uses radioisotopes of the same or chemically similar elements to label biological ligands in a way that allows the use of diagnostic and therapeutic radiation for a combined diagnosis and treatment regimen. For scandium, radioisotopes -43 and -44 can be used as diagnostic markers, while radioisotope scandium-47 can be used in the same configuration for targeted therapy. This work presents the latest achievements in the production and processing of radioisotopes and briefly characterizes solutions aimed at increasing the availability of these radioisotopes for research and clinical practice.

## 1. Introduction

The development of technologies related to nuclear medicine requires providing new solutions in the field of imaging and the production of radioisotopes that can perform specific functions. One of them is the concept of personalized medicine, where the treatment process is matched based on individual screening of tumor phenotypes [1]. In this approach, dedicated compounds with a high affinity for cancer cells are designed based on test results [2]. These compounds can be labeled with diagnostic or therapeutic radioisotopes to obtain information about the development and progression of the disease or the implementation of a therapeutic process [3].

The concept of theranostics is based on the use of radioisotopes of the same or chemically similar elements to label biological ligands in a way that allows the use of diagnostic and therapeutic radiation for a combined diagnosis and treatment regimen. Despite several theranostic pairs being present in clinical practice, theranostic pairs based on the same element are relatively rare [4]. However, when using radionuclides of two different elements, differences in the pharmacokinetic and pharmacodynamic profile could be observed. For scandium, the radioisotopes -43 and -44 can be used as diagnostic markers, while radioisotope scandium-47 can be used in the same configuration for targeted therapy [5]; therefore, theranostic agents that incorporate the matched-pair radionuclides of scandium-43/scandium-47 or scandium-44/scandium-47 would guarantee identical chemistries and pharmacologic profiles.

Another interesting property of the scandium-44 radioisotope is the relatively rare decay consisting of the co-emission of a positron and a gamma quantum. This feature was used in the development and validation of diagnostic methods that, to refine the imaging, use the detection of two gamma quanta originating from positron annihilation in the coincidence mode, supported by the detection of a single gamma quantum, the so-called 3γ positron emission tomography (PET). The work in [6] supports improvement in the resolution and imaging characteristics of PET.

These features mean that the interest in scandium radioisotopes in nuclear medicine applications is significantly increasing [7]. This paper is intended to discuss the latest achievements in the production and processing of radioisotopes and to briefly characterize solutions aimed at increasing the availability of these radioisotopes for research and clinical practice.

## 2. Scandium Radioisotopes

The chemical properties of scandium are similar to the group of lanthanides, while its ionic radius in the range of about 68–74 pm classifies it among the elements with chemical properties most similar to yttrium. In macrocyclic systems, Sc has a coordination number from three to nine and due to its similarity to other M^3+^ radiometals, such as gallium, yttrium, and lutetium, forms complexes with ligands by connecting through oxygen, nitrogen, and halogen. Acid–base equilibria strongly influence the chemical form in which scandium occurs in aqueous solutions. The distribution of individual chemical forms of scandium indicates that, at pH < 4, the hydrated Sc^3+^ cation is the dominant form. Due to the ease of forming hydroxy complexes together with increasing pH, ScOH^2+^ and Sc(OH)_2_^+^ ions appear in the solution. At a pH value around 4, gradual precipitation of Sc(OH)_3_ begins, but other ions present in the solution can influence scandium solubility: acetates increase the solubility, while phosphates reduce via coprecipitation of ScPO_4_ [8]. Depending on the source of literature data, the solubility constant (K_sp_) ranges from 10^−29^ to 10^−33^ and could be used for selective separation of Sc via precipitation [9,10].

Naturally occurring scandium is composed of one stable isotope: scandium-45 [11]. Other scandium radioisotopes are characterized by short half-lives, with only five having half-lives exceeding seconds. Among these five, scandium-43, scandium-44, scandium-47, and scandium-48 exhibit favorable emission profiles for radiopharmaceutical applications. The last of the relatively stable scandium radioisotopes, scandium-46, is a low-energy beta emitter with a half-life of 83.8 days and is complementarily used in basic research. Specifically, scandium-47 and scandium-48 are β^−^ emitters with a half-life of 3.3492 days and 43.67 h, while scandium-43 and scandium-44 are positron emitters with half-lives of 3.891 h and 4.0421 h, respectively [11]. These radioisotopes have shown promising prospects for theranostic systems and may be effectively employed in diagnostic and therapeutic procedures in the realm of nuclear medicine.

## 3. Production of Scandium Radioisotopes

Due to their properties, scandium radioisotopes can be produced in different ways: using cyclotrons, accelerators, or reactors. In the case of cyclotron production, it is possible to use standard proton or deuteron beams with moderate energies available in medical cyclotrons (p,d, 10–20 MeV per particle) or cyclotrons providing α beams.

The starting elements for the production of scandium are calcium, scandium, titanium, and vanadium isotopes. Three of them in their natural form have a promising property, i.e., a very significant dominance of one of the isotopes: natural calcium contains 96.9% calcium-40, natural scandium contains 100% scandium-45, and vanadium is composed of 99.75% vanadium-51. This makes it possible to irradiate naturally occurring materials, which increases availability and reduces costs (see Table 1). The abundance of titanium isotopes is less favorable, where 73.7% is titanium-48, 8.25% titanium-46, 7.4% titanium-47, and just over 5% are isotopes of titanium-49 and -50. Therefore, it is necessary to use separated isotopes enriched in a specific isotope as target materials. This causes a significant increase in target costs (tens or hundreds of EUR/mg) and difficulties in obtaining appropriately enriched separated isotopes. Another issue is the need to reuse the target material; thus, the target processing has to include a recovery step and not introduce any additional impurities. Examples of production methods are presented in Table 1.

Another potentially attractive pathway for the production of scandium radioisotopes is production from a generator by the decay of the parent radionuclide, which is immobilized on the solid phase and then selectively eluted on the scandium radioisotope. It replicates the concepts of technetium-99 m and gallium-68 generators commonly and successfully introduced in nuclear medicine, significantly facilitating the availability of radioisotopes in clinical applications [23]. Scandium-44 is produced as a decay product of titanium-44 formed in the reaction ^45^Sc(p,2n)^44^Ti, while scandium-47 is a decay product of calcium-47 produced in ^46^Ca (n,γ)^47^Ca or ^48^Ca(γ,n)^47^Ca reactions. The status of generator systems for scandium-44 was recently reviewed [23]. In its current state, the concept is very interesting, but it has not been possible to create a generator that would provide enough activity for clinical applications. The first solutions [24] offered 185 MBq of activity suitable for simple preclinical studies, but the eluate required an additional purification step for effective labeling [25]. However, the availability of scandium-44 from the generator enabled a significant development of preclinical work aimed at searching for appropriate macrocyclic ligands for scandium [26]. Work is being carried out to increase the efficiency of the ^44^Ti/^44^Sc generators, significantly increasing the quality of the eluate [27,28,29,30], but at present no generator solution has been presented that could routinely and commonly provide access to the radioisotope similar to technetium or gallium generators.

The idea supporting the development of scandium generators is proof of the concept of the ^47^Ca/^47^Sc generator, which would supplement the theranostic pair with a therapeutic radioisotope from the generator. One approach is to irradiate ^46^Ca (n,γ)^47^Ca- > ^47^Sc. Although the cross-section of this reaction is 740 mb, which is promising, the natural abundance of calcium-46 (0.004%) and low enrichment (~30% ^46^Ca) combined with the ~4000 EUR/mg price of enriched material make this technology extremely expensive. Chemical isolation of scandium-47 from the target material enabled the formulation of up to 1.5 GBq of scandium-47 with high radionuclidic purity (>99.99%) in a small volume (~700 μL) [22]. Another approach is production via photonuclear reaction ^48^Ca(γ,n)^47^Ca- > ^47^Sc, but the effects of irradiation of the ^nat^CaCl_2_ target reached only 1–1.5 MBq of scandium-47 in eluate [31]. Using an enriched calcium-48 target increases results up to tens of MBq activity of scandium-47 [32]. The potential drawback is the relatively short half-life of the produced calcium-47 (T_1/2_ = 4.5 d), resulting in a 10–15-day shelf life of the generator, which poses challenges similar to the logistics of technetium generators.

The possibility of creating complementary ^44^Ti/^44^Sc diagnostic and ^47^Ca/^47^Sc therapeutic generators would be an ideal solution from the point of view of clinical theranostics, but this vision is quite distant yet.

In conclusion, the comparison of scandium radioisotope production methods reveals a wide range of techniques and available hardware platforms. However, a notable challenge lies in the mismatch between cost-effective and readily available target materials, which demand specialized and less common irradiation systems. Conversely, the more widely used irradiation systems necessitate separated materials and, in certain cases, the production conditions for specific radioisotopes are at the limits of the hardware capabilities.

## 4. Radiochemical Separation

### 4.1. Separation and Preconcentration of Cyclotron-Produced Scandium

The methodology for the separation of scandium radionuclides from target material depends strongly on the method utilized for their production. CaO, CaCO_3_, and Ca are used mainly as the target material for cyclotron-produced scandium, thus following the dissolution of these targets in HCl or HNO_3_ solution. The excess of calcium should be removed to the biggest possible extent before synthesis of a radiopharmaceutical. Additionally, Fe, Al, Zn, and Ni (regarded as potential introduced contaminants, e.g., from a target or laboratory equipment) should be eliminated from the final product, as they are important competitors for further scandium labeling of PET molecules.

**Figure 1:**
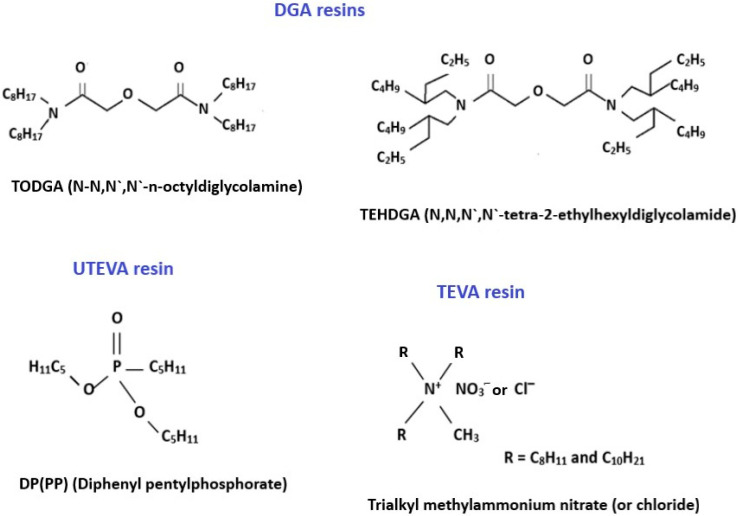
The structures of compounds mostly used in extraction chromatography for Sc separation.

For the separation and purification of Sc radioisotopes from calcium target material, solid-phase extraction (SPE) and extraction chromatography (EXC) are usually employed. EXC methods combine the selectivity of solvent extraction with the ease of operation of column chromatography [33,34]. The extractants for scandium separation are often selected based on the data of the studies of liquid–liquid extraction of this metal as well as other rare earth elements [35,36]. The structures of the Sc active extractants mostly used in extraction chromatography are shown in Figure 1.

**Figure 2:**
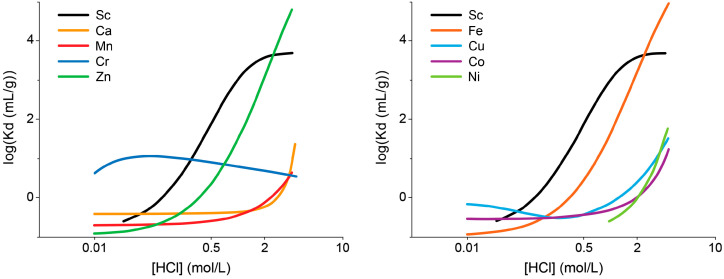
Distribution coefficients (Kd) of some metal ions on TODGA resin as a function of HCl concentration [38].

**Figure 3:**
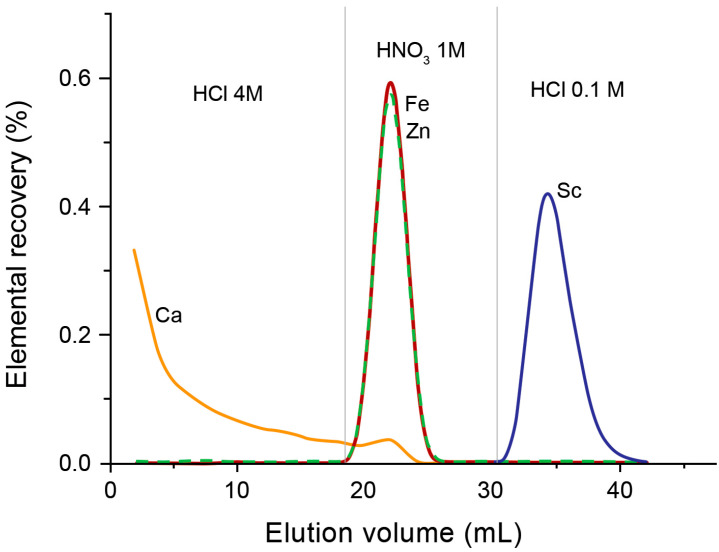
Elution curves for the ^44^Ca irradiated target on TODGA resin [38]. Following the first step in the elution of Ca excess, 12 mL of 1M HNO_3_ was used to elute Fe and Zn. An amount of 10 mL of diluted HCl solution was used to quantitatively elute scandium.

**Figure 4:**
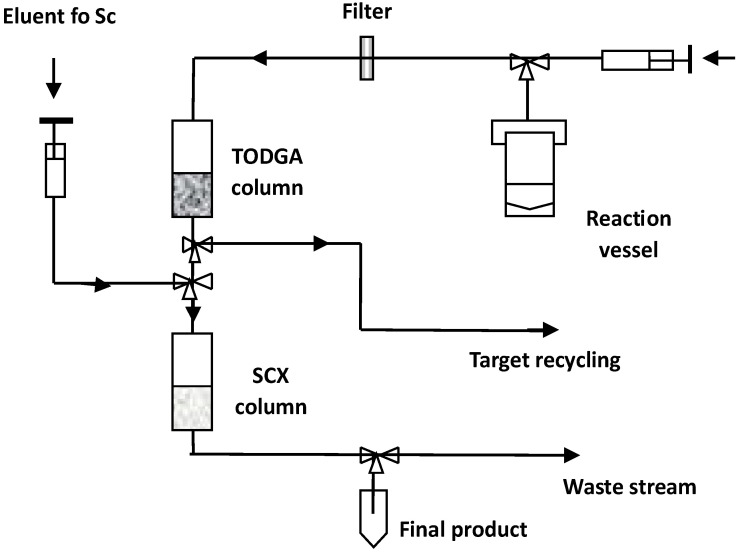
Schematic diagram of scandium-44 production panel using a two-column system [45].

DGA resins, containing *N*,*N*,*N*′,*N*′-tetra-n-octyldiglycolamide (TODGA, also named as normal or non-branched DGA) or *N*,*N*,*N*′,*N*′-tetra-2-ethylhexyldiglycolamide (TEHDGA, named as branched DGA) adsorbed on the surface of hydrophobic polymers, were the most often used to separate trace amounts of Sc from the bulk quantities of calcium [16,20,37,38,39,40,41]. The metal–ligand complexation involves three DGA molecules encapsulated in Sc(III) ions via two carbonyls and an ether oxygen donor [42,43]. To choose the optimum conditions for the purification of Sc, the distribution coefficients (Kd) for that and other metal potential impurities were determined as a function of HCl and HNO_3_ concentrations [36,44]. Sc(III), Zn(II), and Fe(III) ions exhibited a similar and strong affinity for TODGA resin in HCl concentrations > 3 mol/L, as is shown in Figure 2, whereas under these conditions, Ca(II), Mn(II), Cu(II), Co(II), and Ni(II) remained in hydrochloric acid solution. For this reason, irradiated calcium targets were usually dissolved in 3–6 M HCl [16,20,37,38,39,40,45]. The use of HNO_3_ instead of HCl ensured the elution of Fe(III) and Zn(II) from DGA resin and its separation from the desired Sc(III) product [36,37,43]. Alliot et al. [38] proposed a removal procedure for Fe(III) and Zn(II) via elution with 1 M HNO_3_ solution after rinsing the DGA column with 4 M HCl (Figure 3). For the direct labeling and subsequent application in nuclear medicine, preconcentration of Sc radionuclides is necessary in a small volume of solution. In addition, this step allows further reduction of impurities in the final product as well as recovery of expensive enriched calcium targets. Thus, the second DGA column was employed to enable the elution of Sc in a small volume of diluted HCl solution [16]. Also, cation exchange resins, such as Dowex 50WX2 [36,37,39], Dowex 50Wx8 [46], and Bond Elut SCX [16,36], were used for Sc preconcentration in the proposed separation systems. The schematic diagram of the scandium-44 production panel using the two-column system is presented in Figure 4.

After dissolving enriched [^44^Ca]CaCO_3_ in 3 M HCl, scandium-44 was separated from the target material using TODGA resin and eluted with 4 mL of 0.1 M HCl. The solution was passed through the directly connected second column packed with SCX resin for Sc preconcentration. The elution of scandium was performed via a separate valve using 0.7 mL of 5 M NaCl/0.13 M HCl at pH 0–0.5. The ammonium oxalate precipitation method recovered calcium-44 after washing the TODGA column.

**Figure 5:**
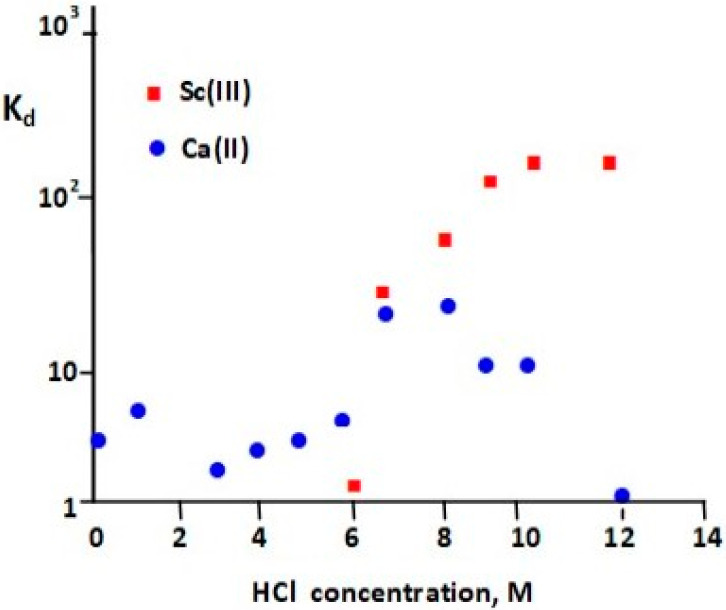
Distribution coefficients of Sc(III) and Ca(II) in UTEVA resin at different HCl concentrations [48].

UTEVA (Uranium and Tetravalent Actinide) resin, as its name suggests, was specially designed for the separation of U(VI) and tetravalent actinide elements, like Np(IV), Th(IV), and Pu(IV) [47]. The extractant coated onto the inert support is dipentyl pentylphosphonate (DP[PP]) (also called diamyl amylphosphonate, DAAP), which is retained on resin via hydrophobic interaction. Its structure, shown in Figure 1, is similar to that of tri-n-butylphosphate (TBP), one of the most common organophosphorus extractants for scandium [46]. Sorption of Sc(III) depends on the concentration of HCl or HNO_3_ in the sample solution and increases with increasing acid concentration. Valdovinos et al. [48] found a greater than a 10-fold difference in distribution coefficients between scandium and calcium at a HCl concentration higher than 9 M (Figure 5). Thus, UTEVA resin was often used for the separation of scandium radioisotopes from Ca targets [12,46,48,49]. The Ca targets are typically dissolved in concentrated HCl and directly loaded onto a UTEVA column. After rinsing the column with the HCl solution, Sc is recovered in a small volume of water in yields typically above 80% and with suitable purity for labeling. The recovery of the expensive Ca target material at high efficiency makes the process cost-effective. The method for separation of Sc radionuclides from Ca targets using UTEVA and the tandem of TODGA + Dowex 50X8 resins were compared concerning Sc recovery, the composition and volume of that fraction, and the possibility of separation from metallic impurities [41].

The effective separation of scandium-43 from calcium target material is possible using both methods, with Sc recovery percentages of 80% for UTEVA resin and 87% for TODGA + Dowex 50X8 resins. The final volume of eluate for the two-step separation (0.65 mL) method containing ammonium acetate buffer can be used directly for labeling and producing radiopharmaceuticals, while separating acidic eluate (0.4 mL in 0.8 M HCl) from UTEVA requires neutralization. However, the level of iron was much lower for UTEVA (<0.001 mg/L) than for the tandem resins (0.56 mg/L).

Another approach to Ca/Sc radiochemical separation is to utilize Chelex 100, a styrene–divinylbenzene copolymer containing paired iminodiacetate functional groups [41,49,50]. In this case, calcium targets were dissolved in less concentrated HCl solutions (0.1–1 M) in comparison with the use of DGA or UTEVA resins. The concentrations of Ca(II) and Fe(III) in scandium fractions were less than 1 mg/L, but Sc recovery was in the range of 79–85%. Walczak et al. [41], after comparing the performance of UTEVA, Chelex, and tandem DGA+ Dowex 50X8 resins, concluded that the best method for isolation of scandium-44 is the use of procedures with UTEVA and Chelex 100 due to the simplicity of the operation. On the other hand, the reported comparison of the Sc separation methods using these two resins and precipitation as Sc(OH)_3_ followed by filtration through a 0.2 µm filter stated that the last procedure is more effective, as it is faster (only 15 min) and more efficient (96% of Sc recovery) [49]. However, Fe concentration in the scandium fraction decreased in the following order: UTEVA extraction resin < filtration < Chelex 100 chelating resin.

Kilian et al. proposed the use of the Nobias Chelate PA1 chelating resin for scandium radioisotope separation and further synthesis of its radiopharmaceuticals [51]. This resin consists of hydrophilic poly(hydroxy methacrylate) beads functionalized with ethylenediaminetriacetic acid and iminodiacetic acid and exhibits a high affinity for rare earth elements, particularly in the saline matrix [52,53].

The [^44^Ca]CaCO_3_ target was dissolved in 1 mL of 2 M HCl, and the obtained solution was adjusted with formic buffer to pH 3 and passed through the column containing 10 mg of resin. The elution of scandium was carried out using 100 µL of 2 M HCl with a (94.9 ± 2.8)% yield. The concentrations of metal impurities were at very low levels, differing favorably from their content in other separation procedures.

**Figure 6:**
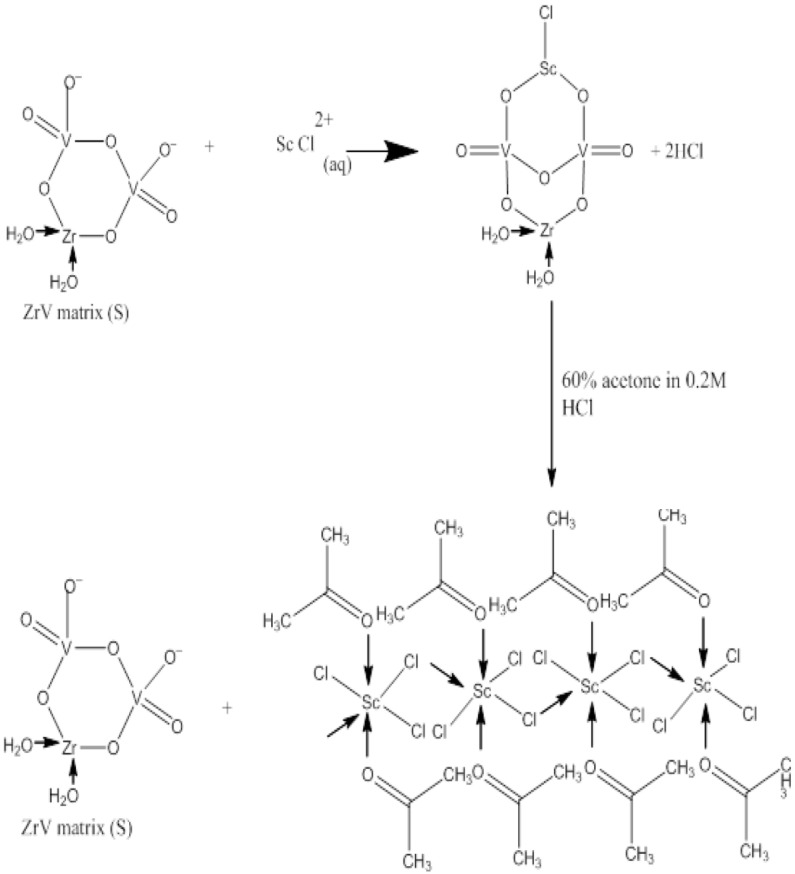
Proposed mechanism for the separation of scandium-47 using the ZrV matrix [54].

Gizawy et al. presented an interesting study regarding the use of zirconium vanadate gel (ZrV) for the effective separation of scandium-47 from the irradiated natural calcium target [54]. This inorganic sorbent was synthesized from zirconium oxychloride and sodium vanadate. The determined Kd values of Sc(III) and Ca(II) ions as a function of HNO_3_ or HCl concentration showed that scandium ions were strongly adsorbed to the ZrV matrix from HNO_3_ or HCl solutions in their concentration in the range of 0.001–0.01M, while Ca was weakly adsorbed. The suggested mechanism for Sc(III) sorption on the ZrV matrix and its elution is presented in Figure 6. Thus, the radioactive tracers of scandium-47 and calcium-47 in 0.001 M HNO_3_ were passed through the column containing 600 mg of the prepared ZrV matrix followed by a 10 mL mixture of 0.2 M HCl with a 60% acetone solution for scandium elution. This method produced a yield of (88 ± 2.2)% and a Ca concentration of 0.05 mg/L in this fraction.

Table 2 summarizes the literature’s reports for the separation of scandium radioisotopes from Ca targets using extraction chromatography and solid-phase extraction.

### 4.2. Separation and Preconcentration of Generator-Produced Scandium

After the production of scandium radionuclides, they need to be separated from macro-quantities of titanium and other impurities. This procedure is usually conducted using SPE or EXC techniques, as in the case of calcium target materials. Sorbents, eluents, and pH are examples of the various parameters studied in these separations [56,57]. A summary of various developed approaches is provided in Table 3. DGA resins were very often used for this purpose [27,37,45,58,59], which is similar to previously described procedures for the separation of scandium from calcium targets. In most cases, they are used in a one-column system. Van der Meulen used a system, similar to that presented in Figure 4, where scandium eluted from TODGA resin using 4.0 mL of HCl 0.1 M is then preconcentrated on the SCX cation exchange resin to a smaller volume (700 µL of 4.8 M NaCl/0.13 M HCl) [37,45].

**Figure 7:**
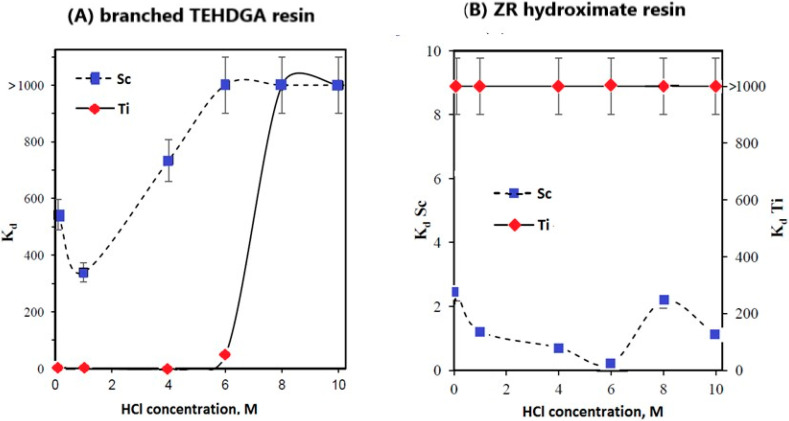
Influence of HCl concentration on the values of K_d_ for ^44^Ti/^44^Sc on: (**A**) TEHDGA resin and (**B**) ZR hydroxamate resin (ZR) [27].

Sc(III) exhibits strong retention at HCl concentrations < 6 M on TEHDGA resin, while under these conditions the retention of Ti(III) was negligible (Figure 7A) [27]. Thus, after the loading step, titanium could be eluted from the column through additional washing with 4 M HCl solution. Unlike TEHDGA resin, titanium strongly adsorbs on the ZR hydroxamate resin, and Kd values higher than 103 were determined to be in the HCl concentration range of 0.1–10 M HCl (Figure 7B).

**Figure 8:**
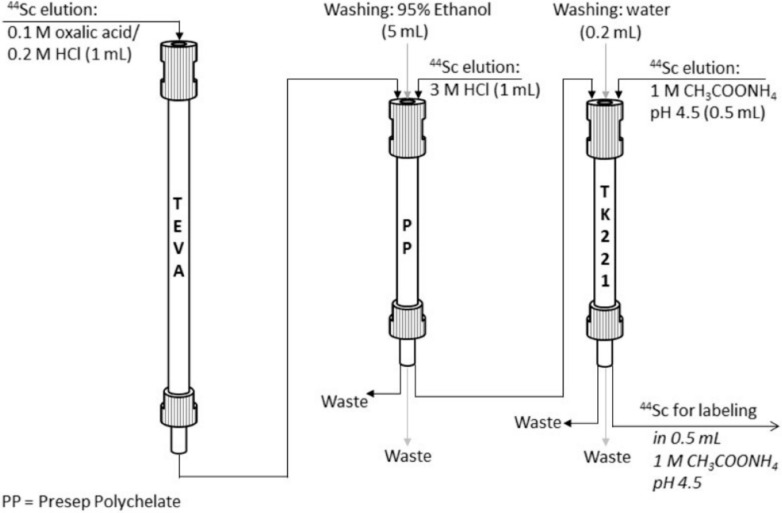
The scheme for TEVA-based ^44^Ti/^44^Sc generator eluate post-processing [28].

Rösch et al. reported that additional washing with HNO_3_ would help to reduce the Fe(III) content for which the stability constant with the most commonly used DOTA ligand is greater than that for Sc(III) [59]. Elution of scandium from TODGA resin was carried out using 0.1 M HCl. The elution profile of titanium-44 and scandium-44 is presented in Figure 8. To increase the efficiency of scandium-43 radiolabeling with the peptide receptor DOTA-NOC, as well as to reduce other impurities, scandium was preconcentrated in a smaller volume with the use of a second column containing SCX cation exchange resin and then eluted with a 4.8 M NaCl/0.1 M HCl solution [37].

Filosof et al. proposed the procedure for separation of scandium-44 from the titanium-44 target using anion exchange resin AG1-X8 because Sc(III) is strongly adsorbed from oxalic acid solution, and its oxalate complex is destroyed by the addition of HCl [17]. The determined distribution coefficients for Ti were higher than 1000 for HCl concentrations < 0.2 M and 0.1 M H_2_C_2_O_4_, while negligible scandium retention was obtained in the presence of different concentrations of HCl/H_2_C_2_O_4_ mixtures. However, it was decided to elute Sc with a lower salt concentration (0.005 M H_2_C_2_O_4_/0.07 M HCl) in the content of further scandium-44 use for radiopharmaceutical synthesis in accordance with the obtained ratio of Kd for Ti to Sc equal to 250 [17]. The conducted experiments showed that Sc(III) did not exhibit strong affinity toward AG 50W-X8 cation exchange resin under these conditions; the ratio of Kd values for Sc to Ti was only 52.

Pruszyński et al. carefully examined the post-elution processing of the ^44^Ti/^44^Sc generator to concentrate and purify the scandium-44 eluate [24]. After passing 0.005 M H_2_C_2_O_4_/0.07 M HCl mixture through the ^44^Ti/^44^Sc generator, the eluted scandium-44 was adsorbed on the small cartridge packed with AG 50W-X8 cation exchange resin. Then, the column was washed with 2–4 mL of water; finally, 5 mL of air was blown through the column. For scandium elution, 3 mL of 0.25 M ammonium acetate solution at pH 4 was applied with 90% of its recovery. This scandium-44 solution has a small volume (2–3 mL) and is free from competing oxalate ions. The cation exchange resin Dowex 50Wx8 was also evaluated for the separation of scandium radionuclides from other elements [60]. After the dissolution of natural TiO_2_ powder in hot concentrated H_2_SO_4_ and later dilution with water, the resulting solution was passed through the column packed with Dowex 50W-X8 resin for adsorption of Sc and Ti. Later, a 2 M HNO_3_ solution was used for washing and releasing Ti(IV); finally, Sc(III) was eluted via the mixture of 4 M HCl and 0.1 M HF solution. However, the proposed separation procedure takes a relatively long time (1 h and 45 min).

Larenkov et al. replaced the earlier proposed AG1-X8 anion exchange resin with TEVA sorbent for the uptake of titanium and successful elution of scandium using the mixture of 0.1 M H_2_C_2_O_4_/0.2 M HCl [28]. Elution of the main scandium-44 activity (95% of all eluted activity) occurred in the first 0.5 mL, which made it possible to achieve a very high separation factor of 1.6 × 10^7^. However, this eluate was not suitable for direct use in radiopharmaceutical synthesis due to the high concentration of oxalic acid. Although decarboxylation using hydrogen peroxide permitted a decrease in the oxalate concentration up to 0.0001 M with scandium-44 recovery of about 87%, this approach was not very convenient for automatization and implementation in a synthesis module. Thus, several different SPE resins were tested for efficient trapping of scandium-44 directly from the generator eluate. As a result of the conducted research, the scheme for TEVA-based ^44^Ti/^44^Sc generator eluate post-processing was proposed, which is depicted in Figure 8. The eluate from TEVA resin was passed through Presep Polychelate sorbent, containing carboxy-methylated polyethyleneimine as a functional group, for quantitative sorption of scandium (>99%). To remove the residues of the sorption solution, the column was washed with 95% ethanol, and then the elution of Sc was carried out using 3 M HCl. However, the content of oxalic acid in this solution was still too high for sufficient radiolabeling despite increasing the volume of the washing water–ethanol solution. Finally, an additional purification step was performed with TK221 resin based on a mixture of a diglycolamide and a phosphine oxide impregnated onto an inert support. The highest scandium-44 recovery was achieved with 1 M CH_3_COONH_4_ (0.5 mL, pH 4.5).

The proposed method takes no more than 15 min, and no titanium-44 was detected in all samples of scandium-44 solutions after this combined post-processing.

## 5. Scandium Complexing Ligands

The almost immediate application of scandium radioisotopes in preclinical studies is the result of the synergy effect due to their significant similarity in properties to the lanthanides and other metallic trivalent elements used in nuclear medicine, as described earlier. This allowed the use of bifunctional ligands (BFCs), which are compounds acting as a conjugate between the radioisotope and a biological vector. The BFC’s key features are the ability to quickly and steadily incorporate the radioisotope and to create covalent bonds with biomolecules responsible for targeting. The trivalent cation Sc^3+^ allows simple labeling with typical bifunctional chelators with tetraaza-ring-like DOTA (1,4,7,10-tetraazacyclododecane-1,4,7,10-tetraaceticacid), TETA (triethylenetetramine) and derivatives (DO3AP (1,4,7,10-tetraazacyclododecane-1,4,7-tri-acetic acid) with different substituents), triaza ring (NOTA (1,4,7-triazanonane-1,4,7-triyl)triacetic acid), and open ligands with nitrogen and oxygen atoms and hydroxylic groups like DTPA (diethylene triamine pentaacetic acid), EDTA (ethylene diamine tetra acetic acid), PYPA (pyridinecarboxylate), DUPA (2-[3-(1,3-dicarboxypropyl)ureido]pentanedioic acid), or HOPO (hydroxypyridinone). In all these cases, a simple recipe consisting of approx. 30 min of labeling at a temperature of 80–90ºC at a pH of about 4 resulted in high labeling efficiency (>80%), high radiochemical purity of the product, and good specific activity. Unfortunately, it did not provide any advantage over existing applications of gallium-68, which is easily obtained from a gallium generator. Recently, a promising bifunctional chelator for Sc with outstanding labeling capabilities at room temperature has been introduced [61]. AAZTA (*N*,*N*′,*N*″,*N*″(6-amino-6- methylperhydro-1,4-diazepine)-tetraacetic acid) shows quantitative complexation with fast kinetics under mild conditions (5 min, room temperature), with better thermodynamic stability than a gallium-68 analog [62]. Successful connections with outstanding labeling capabilities at room temperature with the biological vectors were reported: [^44^Sc]-ScAAZTA5-TOC for management of neuroendocrine tumors [63] and [^44^Sc]-ScPSMA-inhibitor [64,65] and [^44^Sc]-ScPSMA-617 [63] for high-efficiency imaging of prostate cancer. An additional AAZTA feature is high flexibility toward analogs containing phosphate, mono-, and di-glutarate groups or direct links to peptide labeling [66]. All applications featured excellent kinetics with high-yield labeling at room temperature. This opens up an opportunity for effective competition with gallium-68 in the area of the so-called “cold-kit” [67], where ready-made kits are prepared for labeling, similar to technetium-99 m [68]. The approach is quite recent and attractive, which allows for effective competition; a significant part of gallium-68 kits requires several minutes of incubation at an elevated temperature [69].

Some attention has been given to comparing the pharmacological properties of Sc-labeled radiopharmaceuticals with counterparts well established in nuclear medicine. ^44^Sc/^68^Ga-labeled DOTA-NAPamide was produced and used in MC1-R-positive B16-F10 cells and showed significantly (*p* ≤ 0.01) 15-fold higher in vitro radiotracer accumulation in B16-F10 tumor-bearing mice than that of A375 tumors used as reference; this difference was significant using both radiotracers [70]. Comparison of different prostate-cancer-specific ligands, PSMA-617 and PSMA-11 labeled with scandium-44, gallium-68, and lutetium-177, showed that the overall tissue distribution of [^44^Sc]-ScPSMA-617 resembled that of [^177^Lu]-LuPSMA-617 most closely, while the ^68^Ga-labeled ligands, in particular [^68^Ga]-GaPSMA-11, showed slightly different distribution kinetics [71]. AAZTA5-TOC achieved quantitative labeling (>95%) at room temperature in less than 5 min with scandium-44, gallium-68, and lutetium-177 and appears to be a promising bifunctional chelator for all their radioisotopes with outstanding labeling capabilities and high stability [63]. Labeling of PSMA inhibitor with AAZTA as a bifunctional ligand with gallium-68, scandium-44, copper-64, and lutetium-177 resulted in quantitative labeling at room temperature and moderate pH values (4.0–5.5) in all cases, proving the versatility of chelators and radionuclides for instant kit-type labeling [64]. Similar results were obtained for two new targeting vectors based on curcumin scaffolds and linked to the chelators 1,4,7-triazacyclononane,1-glutaric acid-4,7-acetic acid (NODAGA) and AAZTA, where gallium-68 and scandium-44 performed equally [72]. A comparison of gallium-68, scandium-44, and ^177^Lu-labeled AAZTA5-PSMA-617 with DOTA-PSMA-617 analogs showed internalization ratios for the radiolabeled gallium-68, scandium-44, and [^177^Lu]-LuAAZTA5-PSMA-617 tracers (13–20%IA/10^6^ cells) in the same range as the radiolabeled DOTA-PSMA-617 tracers (17–20%IA/10^6^ cells) in the same assay, although lower [^68^Ga]-GaAAZTA5-PSMA-617 stability in human serum, PBS, and EDTA/DTPA solutions was reported [63].

The therapeutic potential of scandium-47 in comparison with lutetium-177 and yttrium-90 was verified in preclinical therapy of ^47^Sc-folate, ^177^Lu-folate, and ^90^Y-folate of folate-receptor-positive ovarian tumor cells. The treatment resulted in increased median survival of 39, 43, and 41 days, respectively, as compared with 26 days in untreated controls [73].

The presented examples show that scandium radioisotopes behave as well as the competitors, gallium-68 and lutetium-177, and can be used interchangeably wherever the field of application is not regulated by the provisions of the pharmaceutical law.

## 6. Imaging Performance of Scandium-43 and -44

Scandium radioisotopes -43 and -44, owing to the kinetic energy of the emitted positron lower than gallium-68, can improve the resolution in the PET imaging process. The proof is the comparison of the quantitative capabilities of diagnostic scandium radioisotopes in commercial PET/CT or microPET devices with other conventional clinical radionuclides (carbon-11, fluorine-18, gallium-68, copper-64, or zirconium-89). Using Derenzo phantoms and a small-animal PET scanner, increasing relative resolution was determined in the sequence of ^68^Ga < ^44^Sc < ^89^Zr < ^11^C < ^64^Cu < ^18^F, in agreement with the theoretical expectations based on the energy of the emitted positrons [74]. The experiment on NEMA phantoms with scandium-43, scandium-44, gallium-68, and fluorine-18 showed that all radionuclides presented similar noise levels, but regarding the background calibration, based on the acceptable deviation of 10% between measured and known background activity concentration, scandium-44 was outside tolerance levels, reaching them with additional corrections. Comparing recovery coefficients, fluorine-18 was superior to all metallic radioisotopes [75]. Direct comparison using Derenzo and NEMA phantoms between gallium-68 and scandium-44 showed that despite high-energy γ-rays in scandium-44 decay (>99.9% 1157 keV), a better image resolution of small structures was observed with scandium-44. Structures as small as 1.3 mm (vs. 1.5 mm for gallium-68) using the Mediso system, and as small as 1.0 mm (vs. 1.3 mm for gallium-68) using the Siemens system, could be visualized [76]. The better image resolution makes scandium-44 an especially strong competitor in preclinical settings because additional strong and intensive γ-emissions have a small impact on the imaging resolution but result in higher background noise, which significantly increases the dose burden in clinical application. The results show that accurate quantitative scandium-43 and scandium-44 PET/CT are achievable in commercial devices.

A curiosity of practical importance for scandium-44 is the tendency to three-photon decay when the third γ is emitted from the nucleus a few ps (2.61 ps for scandium-44) after the β^+^ decay. Scandium-44 has the best properties in terms of availability, β^+^, and gamma-emitted properties and biological–chemical properties for clinical applications among the 17 known radioisotopes with the ability to emit positrons and electrons in the correct sequence [6]. This property is used in extensions of PET modality where the location of the radionuclide is then obtained through the intersection of the third γ direction with the conventional line of response in the PET detector ring. It allows localization of the emission point with event-by-event compensation and helps to refine the measurements by improving the spatial resolution of the detection system. The design work is at the prototype stage [77,78,79,80], but the results could open up new ideas for radioisotope imaging, such as a 3γ full-body scanner with plastic detectors [81].

## 7. Discussion and Further Trends

There exists a significant deficiency in a standardized methodology for radioisotope production and unified target material processing. Initial attempts to address these challenges for scandium radioisotopes -43 and -44 [82] and scandium-47 [83], along with the formulation of comprehensive recommendations [5], represent crucial steps toward integrating scandium-43, -44, and -47 into clinical practice.

Mass production of radioisotopes of scandium faces a critical issue due to the absence of dedicated solutions for routine manufacturing and distribution of scandium radioisotopes. The lack of automatic target material processing raises doubts about its feasibility in clinical settings. Manual processing methods are inadequate due to varying radioisotope quality and the substantial dose burden on operators. Additionally, satisfactory solutions for the application of scandium generators in diagnostics and therapy are still missing.

In clinical practice, PET diagnostics rely on fluorine-18, which benefits from the production scale effect [84]. Fluorine-18 can be efficiently distributed to hospitals through a “satellite model” where one production unit serves multiple centers. This is facilitated by the construction of high-yield synthesizers that allow for multiple production cycles without compromising sterility conditions. However, implementing this model for scandium presents challenges due to expected lower demand, complex isolation processes, and the use of solid targets, which are more challenging to process. Furthermore, competition from gallium-68, established in clinical practice, poses a significant obstacle for scandium-43 and scandium-44.

Exploring methods for producing scandium radioisotopes from common stable isotopes like calcium-40 or natural calcium, supported by the use of automated separation and purification techniques, holds promise due to the low cost and significant enrichment of the target material. While the quest for the ideal production, separation, and application chain for scandium remains elusive, a wide array of production methods, often at reasonable costs, along with effective manual techniques for radioisotope separation, create an excellent opportunity for wide application in preclinical studies. Here, compromises in terms of activity or logistics are permissible. The insights gained from this study can be readily translated to clinical settings through the use of well-established gallium-68 or lutetium-177 radioisotopes.

## Figures and Tables

**Table 1:** Production of scandium radioisotopes.

Isotope	Irradiation Data			Activity	Radionuclidic Purity (%)	Ref.
	Reaction	Abundance (%)	Beam Energy (MeV)			
Target materials with natural isotopic abundance						
Scandium-43	^40^Ca(α,n)^43^Ti- > ^43^Sc	96.9	12.5–17.5	240 MBq/µAh	>98.9	[12]
	^40^Ca(α,n)^43^Ti- > ^43^Sc	96.9	34	54.8 MBq/µAh	99.7	[13]
		96.9	28	102.7 MBq/µAh	99.9	[14]
Scandium-44	^44^Ca(p,n)^44^Sc	2.1	12	6.2 MBq/µAh	N/a	[15]
	^44^Ca(p,n)^44^Sc	2.1	12.8	10.0 MBq/µAh	N/a	[16]
	^45^Sc(p,2n)Ti^44^- > ^44^Sc	100	Generator	185 MBq/elution	N/a	[17]
Scandium-47	^51^V(p,x)^47^Sc	99.75	20–30	1.7 MBq/µAh		[18]
	^51^V(γ,p)^47^Sc	99.75	20	N/a	>99.99	[19]
	^51^V(γ,p)^47^Sc	99.75	38	3.7 GBq	98.2	[19]
Isotopically enriched target materials						
Scandium-43	^46^Ti(p,α)^43^Sc	97	15.1	225 MBq	>98.2	[20]
	^42^Ca(d,n)^43^Sc	93.58	5.8	30.4 MBq/µAh	99.4	[21]
	^43^Ca(p,n)^43^Sc	83.9	13.6	229.0 MBq/µAh	87.8	[21]
Scandium-44	^44^Ca(p,n)^44g^Sc	98.9	13.6	433.3 MBq/µAh	99.7	[21]
	^43^Ca(d,n)^44g^Sc	83.9	5.8	34.4 MBq/µAh	98.1	[21]
Scandium-47	^46^Ca(n, γ)^47^Ca- > ^47^Sc	5.0		2.14 GBq	99.99	[22]
	^47^Ti(n,p)^47^Sc	95.7		4.9 MBq	88–99	[22]

**Table 2:** Separation of scandium radioisotopes from Ca targets using SPE and EXC methods.

Target Dissolution	Separation from the Target		Sc Preconcentration		Recovery of Sc (%)	Impurities (mg/L)	Ref.
	Sorbent	Cleaning	Sorbent	Eluent for Sc			
3 M HCl	TODGA (70 mg)	4 mL of 0.1 M HCl	Dowex 50W-X2 (H^+^ form, 140 µL) Bond Elut SCX (100 mg)	1.5 mL of 0.75 M CH_3_COONH_4_/ 0.2 M HCl (pH 4.5–5.0) 0.7 mL of 5 M NaCl/0.13 M HCl (pH 0–0.5)	98	Ca ~4.5, Pb < 0.7, Al, Zn < 1, Cu < 0.02 (comparable for both resins)	[45]
1 M HNO_3_	TODGA (87 mg)	4 mL of 0.1 M HCl	DGA (43 mg)	700 µL of 0.05 M HCl	Not reported	Traces of ^88^Y	[37]
4 M HCl	TODGA (300 mg)	20 mL of 4 M HCl then 12 mL 1 M HNO_3_	―	10 mL of 0.1 M HCl	88 ± 3	Al + Fe = 1.14	[38]
6 M HCl	TEHDGA (300 mg)	10 mL of 0.1 M HCl	Dowex 50Wx8 (NH_4_^+^ form, 140 mg)	300 µL of 0.1 M NH_4_-α-HIB	95 ± 3	Al 0.06, Fe, Ni 0.03, Zn 0.007, Ca, Ni, Mn < LOD	[16]
3 M HCl	TODGA (70 mg)	4 mL of 0.1 M HCl	Bond Elut SCX (100 mg)	700 µL of 5 M NaCl/ 0.13 M HCl (pH 0–0.5)	90.4 ± 5.5	^88^Y 0.19%	[20]
H_2_O	TODGA (50 mg)	3 times 5 mL of 6 M HCl	―	2.5 mL of 0.05 M HCl	88 ± 6	Ca 6.0, Zn 5.4, Fe 1.1, Pb 0.38, Al 0.18	[39]
4 M HCl	TODGA	10 mL of 1 M HNO_3_	―	0.40 mL of 0.1 M HCl	Not reported	Al + Fe (1.14 ± 0.66)	[40]
3 M HCl	TODGA (70 mg)	2–3 mL of 0.1 M HCl	Dowex 50 (100 mg, H^+^ form)	1 M CH_3_COONH_4_, pH 4	87	Fe 0.56, Ca < 1	[41]
9 M HCl	UTEVA (50 mg)	5 mL of 9 M HCl	―	0.4 mL of H_2_O	79	Fe < 0.001, Ca < 1	
1 M HCl	Chelex 100 (Na^+^ form)	30 mL of 0.01 M HCl	―	0.4 mL of 1 M HCl	85	Fe 10.5, Ca < 1	
9 M HCl	UTEVA (50 mg)	5 mL of 9 M HCl	―	0.40 mL of H_2_O	>80	Fe < 0.001	[42]
12 M HCl	UTEVA (50 mg)	5 mL of 9 M HCl	―	0.40 mL of 1 M HCl	>80	Ca 82.2, Fe 5.2, Zn 4.7, Al 0.17, Ni 1.7, Mn 0.11	[48]
10 M HCl	UTEVA (100 mg)	5 mL of 10 M HCl	―	0.3 mL of H_2_O	>80	Ca, Fe, Zn, Ni, Al, Mn < 0.005	[12]
1 M HCl	Chelex 100	30 mL of 0.01 M HCl	―	2 mL of 1 M HCl	85	Fe 0.99, Ca < 1	[49]
	UTEVA (70 mg)	2 mL of 11 M HCl	AG50Wx4 (H^+^ form)	1 M CH_3_COONH_4_, pH 4.0	93	Not reported	[55]
0.1 M HCl	Chelex 100 (Na^+^ form)	30 mL of 0.01 M HCl	―	1 M HCl	>70	Fe 0.99, Ca < 1	[50]
2 M HCL	Nobias Chelate PA1 (10 mg)	Formic buffer pH 3.0	―	0.1 mL of 2 M HCl	94.9 ± 2.8	Al 0.09, Ca 1.34, Cu 0.02, Fe 0.005, Mn 0.004, Ni 0.013, Pb 1.03, Zn 0.13	[51]
0.01 M HCl	Zirconium vanadate gel (600 mg)	0.001 M HNO_3_	―	0.2 M HCl with 60% acetone	88 ± 2.2	Ca ≤ 0.05	[54]

Abbreviations: DGA—*N*,*N*,*N*′,*N*′-tetra-n-octyldiglycolamide resin; SCX—Strong Cation Exchange; α-HIBA—α-hydroxyisobutyric acid; UTEVA—Uranium and Tetravalent Actinide; DP[PP]—dipentyl pentylphosphonate.

**Table 3:** Separation of generator-produced scandium from Ti target using SPE and EXC methods.

Target Dissolution	Separation from the Target			Recovery of Sc (%)	Impurities	Ref.
	Sorbent	Cleaning	Eluent			
6 M HCl	TEHDGA (5 mL)	4M HCl	0.1 M HCl	Not reported	Not reported	[26]
	Zr resin, hydroxamate groups (1 mL)	―	6 M HCl/0.65 M H_2_O_2_	>94	Not reported	
Concentrated H_2_SO_4_	TODGA	6 M HNO_3_ then 6 M HCl	0.1 M HCl or HNO_3_	>90	As 0.72, Zn 5.3, Fe 3.5, Ti 9.1, Al 0.27, Ca 4.5 (µg)	[59]
NH_4_HF_2_ (200 mg) in 3 mL of 12 M HCl	TEHDGA (110 mg)	7 M HCl, 7 M HNO_3_	10 mL of 0.1 M HCl	94	Fe 1.0, Cu 0.8, Zn 1.3, V 0.5, Al 0.8 (mg/L)	[58]
6 M HCl	AG1-X8 (Cl^−^ form)	1 M HCl	20 mL of 0.005 M H_2_C_2_O_4/_0.07 M HCl	97	Not reported	[17]
6 M HCl	AG50Wx8 (53 mg, H^+^ form)	20 mL of 0.1 M H_2_C_2_O_4/_0.2 M HCl	2–3 mL of 0.25 M CH_3_COONH_4_ (pH 4)	~90	Not reported	[24]
1 mL of hot H_2_SO_4_ concentrated + 50 mg (NH_4_)_2_SO_4_ + 0.1 mL of H_2_O_2_	Dowex 50WX8, (3 g, H^+^ form)	2 M HNO_3_	100 mL of 4 M HCl + 0.1 M HF	>90	Ti 0.05 mg/L	[60]
	TEVA (150 mg)		0.1 M H_2_C_2_O_4_/ 0.2 M HCl	95		[28]

Abbreviations: TODGA—*N*,*N*,*N*′,*N*′-Tetraoctyl diglycolamide, TEHDGA—*N*,*N*,*N*′,*N*′-tetra-2-ethylhexyl-3-oxopentane-1,5-diamide.

## Data Availability

Data available in the publicly accessible repositories.
